# P-glycoprotein associated with diabetes mellitus and survival of patients with pancreatic cancer: 8-year follow-up

**DOI:** 10.1590/1414-431X202010168

**Published:** 2020-10-07

**Authors:** Nan Hu, Hui Wang, Qing Qian, Yan Jiang, Jun Xie, Dachuan Zhang, Qing Li, Sulan Zou, Rong Chen

**Affiliations:** 1 Department of Pharmacy, The Third Affiliated Hospital of Soochow University, Changzhou, Jiangsu, China; 2 Department of Pathology, The Third Affiliated Hospital of Soochow University, Changzhou, Jiangsu, China

**Keywords:** Cohort study, Cytochrome P450 3A4, P-glycoprotein, Pancreatic cancer with diabetes mellitus

## Abstract

Diabetes mellitus (DM) has a high prevalence in patients with pancreatic cancer (PaC), but the prognostic value of DM in PaC remains controversial. Alterations of P-glycoprotein (P-gp) and cytochrome P450 3A4 (CYP3A4) contribute to multidrug resistance and intestinal metabolism in a variety of cancer types, which may be implicated in DM development. This study aimed to explore the potential prognostic value of P-gp and CYP3A4 in PaC patients in the context of DM through long-term follow-up. We retrospectively reviewed the medical records of patients with PaC admitted at The First People's Hospital of Changzhou, Jiangsu, China, from January 2011 to November 2019 and identified two cohorts of adult patients with PaC, including 24 with DM and 24 without DM (non-DM). The baseline clinical characteristics and outcomes were compared. Immunohistochemistry showed that protein expression of P-gp, but not CYP3A, in duodenum tissues was significantly upregulated in PaC patients with DM compared with those without DM. Kaplan-Meier analysis and log-rank test showed that the survival of patients with PaC and DM/high expression of P-gp was not significantly reduced compared with that of patients without DM/low expression of P-gp. These findings suggested that P-gp expression levels were different in the DM and non-DM groups of patients with PaC, but DM and duodenal P-gp levels were not associated with the long-term survival of patients with PaC. It appears that the presence of DM or P-gp expression levels may not serve as effective prognostic markers for PaC.

## Introduction

According to GLOBOCAN 2018, pancreatic cancer (PaC) is the 11th most common cancer, estimated to have caused 432,242 deaths in 2018 (1). Currently, available treatment options for PaC are surgical resection, chemotherapy, and radiotherapy (2), among which surgical resection is considered the mainstay of curative treatment and is associated with better long-term survival (3). Nevertheless, 80-90% of patients with PaC are diagnosed at an advanced stage and with unresectable tumors, and the 5-year survival rate of PaC is as low as 5% (4). Identification of poor prognostic factors in PaC may provide valuable prognostic information for selecting appropriate treatment protocols. Although factors like tumor stage, grading, lymph node ratio, surgical margin, and tumor size have been identified as prognostic factors in PaC (5,6), it is of great importance to identify new biological or pathological indicators related to the survival of patients with PaC.

Diabetes mellitus (DM) is a group of metabolic disorders characterized by hyperglycemia resulting from insufficient insulin secretion, resistance to insulin action, or both (7), posing an increasingly serious threat to human health worldwide with an estimated global prevalence of 592 million by 2035 (8). DM is known as a possible risk factor and/or an early manifestation of PaC, being concurrently present in 50-80% of patients with PaC (9,10). Accumulating evidence suggests that concurrent diagnosis of DM is associated with increased risk of cancer recurrence and mortality in patients with colorectal, breast, liver, endometrial, and gastric cancers as well as leukemia (11). Nevertheless, how DM affects the clinical outcome and survival of patients with PaC remains controversial. Some studies reported that the presence of DM does not have a statistically significant effect on the overall survival (OS) or the mortality of PaC (11,12), whereas others found a significant association between DM and reduced survival in PaC (13). Therefore, the characterization of a reliable indicator related to survival and clinical outcome is required for predicting prognosis and selecting appropriate therapeutic strategies for patients with PaC and DM.

Systemic chemotherapy is used for advanced PaC after surgery or unresectable metastatic PaC, which significantly extends OS and improves patient outcome (14). Intrinsic or acquired resistance to chemotherapy remains the major cause of treatment failure (15). The drug efflux protein P-glycoprotein (P-gp) and drug-metabolizing enzyme cytochrome P450 3A4 (CYP3A4) are both expressed in the intestinal mucosa and serve as barriers to oral drug delivery by regulating pharmacokinetic and pharmacodynamic interactions during the process of drug absorption and metabolism (16,17). P-gp and CYP3A4 are overexpressed in a number of different cancers such as ovarian, breast, and colon cancers, and some studies showed that they are associated with resistance to therapy and/or poor prognosis (18,19). In patients with coexisting PaC and DM, alterations of P-gp and CYP3A4 may become more complicated due to the hyperglycemic environment and diabetes medications (20). A recent study showed that the expression of intestinal P-gp was significantly increased with the progression of diabetes in rats (induced by low dose streptozotocin and high-fat diet), resulting in a significant decrease in the intestinal uptake and peroral bioavailability of the P-gp/CYP substrates verapamil and atorvastatin (21). Peroral bioavailability of drugs is a primary concern for patients with coexisting PaC and DM who are usually prescribed with multiple drugs for effective management of blood glucose levels and cancer progression. Little is known with respect to the expression patterns of intestinal P-gp and CYP3A4 in PaC patients in the context of DM, as well as their correlation with patient prognosis.

In the present study, we retrospectively reviewed the records of patients with PaC admitted to The First People's Hospital of Changzhou, Jiangsu, China from January 2011 to November 2019, and identified 24 patients with PaC and DM and 24 with PaC but without DM. The protein expression of P-gp and CYP3A4 in the duodenum tissue of each patient was determined. The association between P-gp/CYP3A expression and the presence of DM as well as between survival time and DM presence/P-gp expression were assessed. Our findings may provide a new understanding of the value of intestinal P-gp expression in evaluating the prognosis in PaC patients with DM.

## Material and Methods

### Patients

From the database of patients at The First People's Hospital of Changzhou, Jiangsu, China, from January 2011 to November 2019, we identified 24 consecutive adult patients with pathologically confirmed PaC and DM. Another cohort of unmatched 24 patients with PaC but without DM (non-DM) was randomly selected from the database. The patients with other cancers, undergoing radiotherapy/chemotherapy, with incomplete data, or lost to follow-up were excluded. The patient characteristics are described in Table 1. This study was approved by the Ethics Committee of The First People's Hospital of Changzhou. Informed consent was obtained from each patient.

**Table 1:** Clinicopathological characteristics of patients with pancreatic cancer with or without diabetes mellitus (DM).

Clinicopathological variables	DM (n=24)	Non-DM (n=24)	P
Sex			0.019
Male	10 (41.7%)	18 (75.0%)	
Female	14 (58.3%)	6 (25.0%)	
Age	67.50 (43.00, 83.00)	65.00 (48.00, 82.00)	0.297
Weight (kg)	59.00 (45.00, 78.00)	62.00 (46.00, 89.00)	0.260
Height (cm)	161.50 (152.00, 175.00)	166.50 (156.00, 177.00)	0.032
Smoking/alcohol	1 (4.3%)	3 (12.5%)	0.317
Hypertension*	9 (37.5%)	9 (37.5%)	1.000
Duration of DM (years)	3.00 (0.10, 30.00)	-	N/A
Medications			N/A
N/A	2 (11.8%)	-	
Gliclazide	1 (5.9%)	-	
Metformin	1 (5.9%)	-	
Metformin + repaglinide	2 (11.8%)	-	
Metformin + insulin	1 (5.9%)	-	
Repaglinide	2 (11.8%)	-	
Insulin	8 (47.1%)	-	
Fasting blood glucose (mM)	7.95 (3.80, 12.50)	5.70 (3.90, 12.30)	0.001
Postprandial blood glucose (2 h, mM)	14.90 (6.90, 28.50)	-	N/A
WBC count (×10^9^)	5.86 (3.99, 11.30)	5.27 (3.07, 10.75)	0.068
RBC count (×10^12^ )	3.94 (2.70, 5.18)	4.36 (2.41, 5.41)	0.155
Hemoglobin (g/L)	124.50 (86.00, 160.00)	132.50 (65.00, 149.00)	0.458
ALT (U/L, median, range)	151.50 (7.00, 339.00)	68.00 (11.00, 950.00)	0.773
AST (U/L, median, range)	62.00 (7.00, 259.00)	53.50 (13.00, 883.00)	0.695
Potassium (mM)	4.26 (2.70, 5.66)	4.25 (3.36, 5.55)	0.643
Sodium (mM)	138.10 (132.00, 143.90)	141.15 (135.90, 147.50)	0.003
Chloride (mM)	98.35 (92.00, 106.10)	99.60 (93.70, 107.70)	0.143
Albumin (g/L)	35.35 (28.30, 47.50)	32.40 (22.70, 42.60)	0.101
BUN (mM)	4.24 (2.37, 7.29)	4.32 (1.47, 6.57)	0.386
Creatinine (μM)	71.65 (39.90, 93.00)	63.50 (37.00, 118.20)	0.115
Total cholesterol (mM)	5.32 (2.80, 8.88)	4.08 (2.34, 6.00)	0.002
Triglycerides (mM)	2.28 (0.51, 9.38)	1.69 (0.62, 4.32)	0.334
HDL (mM)	0.92 (0.42, 3.01)	0.96 (0.17, 2.04)	0.599
LDL (mM)	2.41 (0.91, 4.30)	1.97 (0.36, 2.68)	0.045
Tumor stage			0.655
I	4 (16.6%)	2 (8.3%)	
II	16 (66.7%)	19 (79.2%)	
III	4 (16.7%)	3 (12.5%)	
ECOG			0.855
0	5 (20.8%)	7 (29.2%)	
1	14 (58.4%)	13 (54.2%)	
2	5 (20.8%)	4 (16.6%)	
Radiotherapy	2	1	>0.99
Chemotherapy			
Gemcitabine + capecitabine	1	3	0.609
Gemcitabine + oxaliplatin	2	1	>0.99
Gemcitabine +TGOPC	2	1	>0.99
Capecitabine + irinotecan	1	0	>0.99
Gemcitabine	3	2	>0.99
TGOPC	0	1	>0.99
P-gp			<0.001
<85	6 (25.0%)	18 (75.0%)	
≥85	18 (75.0%)	6 (25.0%)	

Categorical data are reported as number (%) and continuous data as median (range) [chi-squared test (categorical) and Student's *t*-test (continuous)]. *Defined as drug treatment for hypertension. N/A, not available; WBC, white blood cell; RBC: red blood cells; ALT, alanine aminotransferase; AST, aspartate aminotransferase; BUN, blood urea nitrogen; HDL, high-density lipoprotein cholesterol; LDL, low-density lipoprotein cholesterol; ECOG: Eastern Cooperative Oncology Group; P-gp: P-glycoprotein; TGOPC: tegafur gimeracil oteracil potassium capsule.

### Immunohistochemistry (IHC)

IHC was performed using the EliVision^TM^ method (Maixin-Bio, Fuzhou, China). Duodenum tissue samples were obtained during surgery and fixed in 4% formalin. Paraffin-embedded tissue sections (3-4-µm thick) were prepared. The sections were dewaxed in xylene and dehydrated in ethanol, followed by incubation with 3% H_2_O_2_ for 15 min. After additional incubation with 10% normal bovine serum for 10 min, each slide was incubated with primary antibodies against human P-gp (MAB-0237, MXB Biotechnologies, China) or CYP3A4 (ab3572, Abcam, UK) at 4°C overnight. The protein expression was visualized using an ultraView universal DAB detection kit (Ventana, Roche, USA). The positive and negative controls were provided by the manufacturer. The results were blindly scored (0-100%) by two independent pathologists using an Olympus IX73 microscope (Olympus Corp., Japan) and the following algorithm: [(3 × intensity of specific immunodetection) + (2 × amount of immunodetected structures) + (2 × intensity of non-specific immunodetection) + (1 × intensity of contrast) + (1 × preservation of morphology)] × 3703 (constant that allows conversion to the 0-100 score), as previously described (22). Five randomly selected fields were scored in each slide. The final score is reported as the average of the scores of two pathologists. A reassessment was performed when the deviation was ≥20%.

### Statistical analysis

Statistical analyses were carried out using the SPSS software (version 19.0; IBM, USA). Data are reported as median (range). Comparison between two groups was conducted using the chi-squared test or unpaired Student's *t*-test. Differences between categorical variables were compared using the chi-squared test. The survival of patients with PaC was assessed using Kaplan-Meier analysis and the log-rank test. A value of P<0.05 was considered statistically significant.

## Results

### Comparison of clinicopathological characteristics between patients with PaC with/without DM

To identify the possible features of DM in patients with PaC, we compared the clinicopathologic characteristics of patients with PaC with/without DM. In the DM group, the median duration of DM was 3.0 (range, 0.1-30.0) years. As shown in Table 1, the blood levels of fasting blood glucose (P=0.001), sodium (P=0.003), total cholesterol (P=0.002), and low-density lipoprotein cholesterol (P=0.045) were significantly higher in the DM group than in the non-DM group, suggesting dysregulated glucose and lipid metabolism in patients with PaC and DM. In addition, the DM group included more women (58.3 *vs* 25.0%, P=0.02), and the patients in the non-DM group were taller (P=0.03), without a difference in weight (P=0.26). There were no differences between the two groups regarding smoking, alcohol, other biochemical indicators, tumor stage, and Eastern Cooperative Oncology Group (ECOG) score (all P>0.05). The proportion of patients with a P-gp score ≥85 was higher in the DM group than in the non-DM group (75.0 *vs* 25.0%, P<0.001).

### P-gp, but not CYP3A4, was significantly upregulated in duodenum tissues of patients with PaC and DM

**Figure 1:**
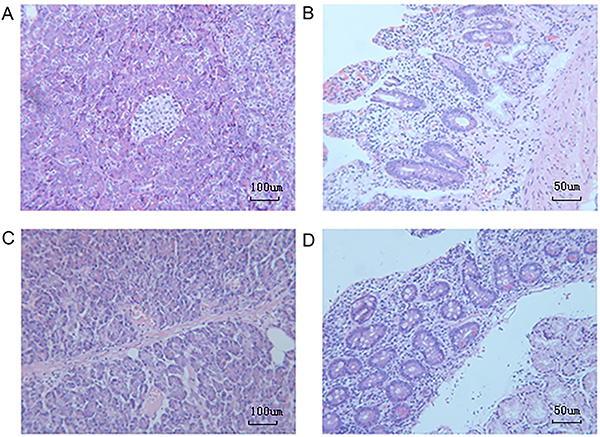
Hematoxylin and eosin staining of duodenum tissues from patients with pancreatic cancer with (**A** and **B**) or without (**C** and **D**) diabetes mellitus. Representative images are shown. Magnification 100× (**A** and **C**, scale bar 100 μm) and 200× (**B** and **D**, scale bar 50 μm).

**Figure 2:**
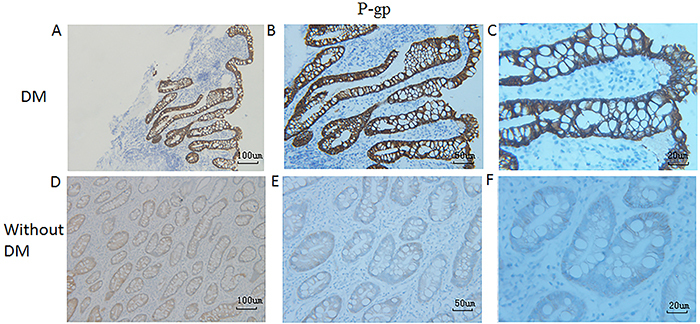
Immunohistochemical staining of P-glycoprotein (P-gp) in duodenum tissues of patients with pancreatic cancer with (**A**, **B**, and **C**) or without (**D**, **E**, and **F**) diabetes mellitus (DM). Representative images are shown. Magnification 100× (**A** and **D**, scale bar 100 μm), 200× (**B** and **E**, scale bar 50 μm), or 400× (**C** and **F**, scale bar 20 μm).

**Figure 3:**
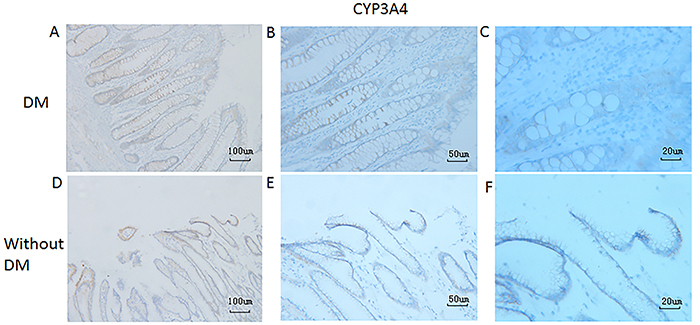
Immunohistochemical staining of cytochrome P450 3A4 (CYP3A4) in duodenum tissues of patients with pancreatic cancer with (**A**, **B**, and **C**) or without (**D**, **E**, and **F**) diabetes mellitus (DM). Representative images are shown. Magnification 100× (**A** and **D**, scale bar 100 μm), 200× (**B** and **E**, scale bar 50 μm), or 400× (**C** and **F**, scale bar 20 μm).

Since P-gp and CYP3A4 are able to form an intestinal absorption barrier that is closely associated with multidrug resistance (17), we sought to investigate the possible association of P-gp and CYP3A4 expression with the presence of DM in patients with PaC. Based on the IHC results for these two proteins in duodenum tissue samples of patients with PaC with/without DM (Figure 1), the mean IHC score of duodenal P-gp in the DM group was significantly higher than that in the non-DM group (Table 2; Figure 2A-F), whereas the mean IHC score of duodenal CYP3A4 was not significantly different between the two groups (Table 2; Figure 3A-F).

**Table 2:** Duodenal expression score (1-100) of CYP3A4 and P-gp proteins in patients with pancreatic cancer with or without diabetes mellitus (DM).

	DM (n=24)	Non-DM (n=24)	P
P-gp	92.50 (20.00, 100.00)	65.00 (10.00, 95.00)	<0.001
CYP3A4	80.00 (20.00, 100.00)	72.50 (0.00, 100.00)	0.312

Data are reported as median (range) (Student's *t*-test). CYP3A4: cytochrome P450 3A4; P-gp: P-glycoprotein.

### DM and duodenal P-gp levels were not associated with the OS of patients with PaC

**Figure 4:**
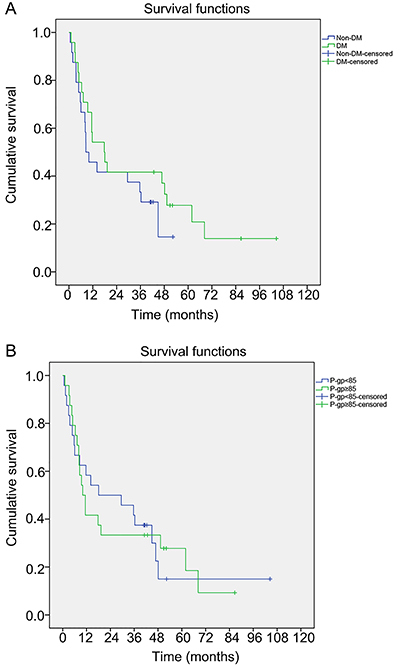
Kaplan-Meier analysis of survival time in patients with pancreatic cancer with or without diabetes mellitus (DM) (P=0.284) (**A**) and duodenal P-glycoprotein (P-gp) score < or ≥ 85 (P=0.981) (**B**).

The OS of patients with PaC and DM or high expression of P-gp (IHC score >85) was not significantly lower compared with that of patients without DM or with low expression of P-gp (P=0.291 and P=0.958) (Figure 4). In addition, the multivariable Cox regression analysis showed that there was no association between other variables and survival in patients with PaC (data not shown). These data suggested that, at least in this cohort of patients, the presence of DM and high duodenal P-gp levels were not associated with the OS of patients with PaC.

### Cox analysis for death

The univariate and multivariate Cox analyses were conducted. Triglycerides (HR=0777, 95%CI: 0.616-0.980, P=0.033) and ECOG=2 (HR=3.047, 95%CI: 1.098-8.459, P=0.032) were associated with mortality in the univariate analyses. In the multivariate analysis, no variable was associated with mortality.

## Discussion

In the present study, we compared the protein levels of P-gp and CYP3A4 in duodenum tissues between patients with PaC with/without DM and found that P-gp, but not CYP3A4, was significantly upregulated in the DM group *vs* the non-DM group, suggesting a possible positive correlation between duodenal expression of P-gp and the presence of DM in patients with PaC. Despite the negative results of the association analyses of patient survival with DM and duodenal P-gp levels, our findings may provide new clues for a better understanding of the chemoresistance-related genetic alterations in PaC in the context of DM.

P-gp protein expression was different in the DM and non-DM groups, suggesting that the expression of the P-gp protein was affected by DM and that drug resistance might theoretically be different. Although P-gp was first identified in drug-resistant cancer cells, it is also expressed in a wide variety of normal tissues (small intestine, liver, and kidney) as well as in blood-tissue barriers (23), serving as an efflux transporter against the entry of toxic xenobiotics, such as therapeutic drugs, dietary compounds, and environmental toxins, into the tissues (24). Previous studies suggest that the expression of P-gp changes in various pathological conditions at different tissue levels (25,26). With reference to DM, Yeh et al. (25) reported that hyperglycemia suppresses renal P-gp expression in rats, whereas another group has observed the overexpression of P-gp in the blood-brain barrier of streptozotocin-induced diabetic rats (27). As peroral bioavailability of drugs will be primarily affected by intestinal P-gp and patients with PaC and DM are usually prescribed multiple medications, it is of great importance to evaluate the intestinal P-gp expression in these patients. In this study, the upregulation of duodenal P-gp expression in patients with PaC and DM was higher than that in patients with PaC but without DM, which was consistent with a recent study showing that intestinal P-gp can be significantly increased along with the progression of DM in rats (21). As an ATP-mediated transporter, the ATPase enzyme is required for the function of P-gp (28), but we did not measure the ATPase activity in this retrospective cohort study, which should be addressed in the future.

Elevated expression of P-gp in response to chemotherapy has been reported in different malignancies, and P-gp was first identified in chemotherapy-resistant cancer cells. For example, a dramatic increase in P-gp expression from 15 to 43% was observed in breast tumor biopsies following treatment with conventional chemotherapy (29). Likewise, in a multiple myeloma study, P-gp expression was 6% at diagnosis, which increased to 43% after treatment (30). In addition, the expression of P-gp is associated with the survival of patients with cancer. Indeed, a shorter OS in patients with acute myelogenous leukemia was associated with upregulated P-gp expression (31). Similarly, patients with PaC and high P-gp expression in PaC tissues have shorter survival compared with those with weak or moderate expression of P-gp (32). Paradoxically, in the same report, it was also observed that the survival of patients with high expression of P-gp was not significantly different from that of those without detectable P-gp expression (32).

Therefore, based on the association between duodenal P-gp levels and DM in patients with PaC, we explored whether DM or duodenal P-gp expression could affect the survival of patients with PaC. We observed shorter survival in the DM and high P-gp expression groups compared with the non-DM and low P-gp expression groups, but the differences between the groups were not significant, possibly due to the small sample size of this study and the impossibility of adjusting for confounding factors. Furthermore, most of the patients in this study were treated with metformin, the first-line drug for type-2 diabetes (33). In addition to its hypoglycemic effects, metformin has been shown to inhibit chemotherapy resistance in a variety of solid tumors, including PaC (34-36). The mechanisms include decreased microvessel density, leakage, and hypoxia (34), enhancement of the effects of anti-proliferation drugs (35), and inhibition of chemoresistance (36). Those effects could improve the survival of the patients and might have contributed to the lack of a significant difference between the DM and non-DM groups in the present study. Indeed, metformin is associated with improved survival in patients with solid tumors (37-40). Future studies should seek to include patients with PaC and DM but without treatment for DM with metformin.

The primary limitation of the present study is that it had a small sample size from a single center. In addition, it was a retrospective cohort study, resulting in selection biases. The data that could be analyzed were limited to those available from the medical charts. Furthermore, based on the available data, it was impossible to determine the causal relationship between DM and PaC with any certitude. Thus, the prognostic value of DM and P-gp in PaC remains disputable (11-13), and a larger sample size may be required for further confirmation of the results of the present study.

In conclusion, our study showed that the protein levels of duodenal P-gp were significantly elevated in PaC patients with DM compared with those without DM. Although there was no significant difference between patient survival and DM or P-gp levels, our results may still be helpful for a better understanding of drug resistance-related gene dysregulation in PaC patients.
